# Gonococcal urethritis caused by a multidrug resistant *Neisseria gonorrhoeae* strain with high-level resistance to spectinomycin in China

**DOI:** 10.1080/22221751.2020.1732836

**Published:** 2020-03-02

**Authors:** Shao-Chun Chen, Li-Hua Hu, Xiao-Yu Zhu, Yue-Ping Yin

**Affiliations:** a Institute of Dermatology, Chinese Academy of Medical Sciences & Peking Union Medical College, Nanjing, People’s Republic of China; b National Center for STD Control, China Center for Disease Control and Prevention, Nanjing, People’s Republic of China; c Zhejiang Provincial Institute of Dermatology, Deqing, People’s Republic of China

**Keywords:** *Neisseria gonorrhoeae*, spectinomycin, resistance, urethritis, treatment failure

## Abstract

We report a recent (2018) gonorrhoeal urethritis caused by a multidrug-resistant *Neisseria gonorrhoeae* strain in China. The isolated *N. gonorrhoeae* strain from a male and female pair expressed high-level resistance to spectinomycin (SPC), azithromycin, and other antibiotics but was sensitive to ceftriaxone. The SPC high-level resistance (MIC = 2048 mg/L) was due to a small deletion in *rspE* that caused two amino acid changes in ribosomal protein S5.

*Neisseria gonorrhoeae* has become resistant to all antibiotics previously used for gonorrhoea treatment, compromising our ability to effectively control gonococcal infections. In China, ceftriaxone (CRO) 250 mg or spectinomycin (SPC) 2 g is used as first-line monotherapy treatment options for urogenital gonorrhoea [1]. However, an increasing level of decreased susceptibility of gonococcal strains to CRO and clinical resistant strains to third-generation cephalosporins (cefixime and CRO) have been reported in recent years [2]. Due to such resistance, SPC is frequently used in empiric therapy of gonorrhoea in many Chinese clinics [3]. Importantly, SPC resistance expressed by gonococci isolated in China is rare making SPC an appropriate replacement antibiotic for the treatment of cephalosporin-resistant cases of gonorrhoea. We now describe a gonorrhoeal urethritis case caused by a high-level SPC-resistant strain that is also high-level resistant to multiple antibiotics.

The patient with an SPC-resistant gonorrhoeal infection was a heterosexual male in his early 40s. He presented at Zhejiang Provincial Institute of Dermatology in August 2018 with urethral discharge and dysuria two days after unprotected sex with a female sex worker. A urethral swab was collected for laboratory diagnosis of gonorrhoea and Gram-negative diplococci were observed within leukocytes by microscopy. He was diagnosed to have gonorrhoea and empirically treated with a single dose of 2 g of SPC (Lukang Pharmaceutical, Shandong, China) intramuscularly and 100 mg doxycycline (Yung Shin Pharm. Ind. Co., Ltd, Jiangsu, China) orally twice a day for five days. As his urethral discharge was not resolved and his wife had pain and discharge at her urogenital tract, he and his wife visited the same STD clinic 10 days later after his first visit. A urethral swab from the male patient and an endocervical swab from his wife were collected and cultured on Thayer–Martin selective media (BioMérieux, Shanghai, China). Although few leukocytes were found from both samples by microscopy, results from a nucleic acid amplification test (Acon, Zhejiang, China) on both samples were positive for *N. gonorrhoeae*. Considering the possible SPC treatment failure may have occurred at the first visit of the male patient, an increased dose of SPC 4 g intramuscularly was used to treat the couple. A telephone follow-up one week after treatment revealed that they both were asymptomatic, but a follow-up test of cure was not performed.

The bacterial samples from the infected male (pre- and post-treatment isolates) and female (pre-treatment isolate) were transferred to the reference laboratory at the National Center for Sexually Transmitted Disease Control (NCSTDC), Chinese Center for Disease Control and Prevention. *N. gonorrhoeae* species were confirmed for all three isolates by Gram stain and a carbohydrate (glucose, maltose, lactose, sucrose prepared at NCSTDC) utilization test. The agar dilution method was used to determine the antibiotic susceptibility of the gonococcal isolates, which included SPC, azithromycin (AZM), penicillin (PEN), tetracycline (TET), ciprofloxacin (CIP) and CRO (USP Reference Standard, Rockville, MD, US). Production of penicillinase was assessed using a nitrocefin solution filter paper test [4]. All three isolates had the same antimicrobial susceptibility profile. Briefly, the strains were high-level resistant to SPC (MIC = 2048 mg/L), AZM (MIC > 256 mg/L), PEN (MIC > 256 mg/L, penicillinase-positive), TET (MIC > 64 mg/L) and resistant to CIP (MIC = 4 mg/L), but susceptible to CRO (MIC = 0.015 mg/L) according to susceptibility and resistance breakpoints from the European Committee on Antimicrobial Susceptibility Testing (Table 1).

**Table 1.** Phenotypic and genetic characteristics of three *N. gonorrhoeae* isolates with high-level resistance to SPC identified in China.

Strains	MIC (mg/L, resistance)							Genotype				Resistant determinants		
	SPC	AZM	PEN	CRO	TET	CIP	PPNG	MLST	*porB*	*tbpB*	NG-MAST	Ribosomal protein 5S (coded by *rpsE*)	16S rRNA	23S rRNA
Male pre-treatment isolate	2048 (R)	>256 (R)	>256 (R)	0.015 (S)	>64 (R)	4 (R)	Yes	7822	4	75	ST304	27 V deletion K28E alteration	WT	A2059G
Male post-treatment isolate	2048 (R)	>256 (R)	>256 (R)	0.015 (S)	>64 (R)	4 (R)	Yes	7822	4	75	ST304	27 V deletion K28E alteration	WT	A2059G
Female pre-treatment isolate	2048 (R)	>256 (R)	>256 (R)	0.015 (S)	>64 (R)	4 (R)	Yes	7822	4	75	ST304	27 V deletion K28E alteration	WT	A2059G

Notes: ^a^SPT, spectinomycin; AZM, azithromycin; PEN, penicillin; CRO, ceftriaxone; TET, tetracycline; CIP, ciprofloxacin; PPNG, penicillinase-producing *N. gonorrhoeae*; ST, sequence type; NGMAST, *N. gonorrhoeae* multi-antigen sequence type; MLST, multilocus sequence type; V, valine; K, lysine; E, glutamic acid; A, alanine; G, glycine; WT, wild type. ^b^Susceptibility (S) and resistance (R) were determined based on breakpoints from the European Committee on Antimicrobial Susceptibility Testing (http://www.eucast.org/clinical_breakpoints). ^c^Reference gene (GenBank accession no. KC311362.1) numbering in the ribosomal protein 5S of *N. gonorrhoeae* was used.

Multilocus sequence typing (MLST) [5], *N. gonorrhoeae* multi-antigen sequence typing (NG-MAST), [6] and *N. gonorrhoeae* sequence typing for antimicrobial resistance (NG-STAR) [7] were performed to identify the sequence type and resistant determinants. All three isolates were assigned to MLST7822 and NG-MAST ST304. The NG-STAR type was a new ST, which contained a type 43 non-mosaic *penA* allele (*penA* 43.002) as well as previously described mutations in other chromosomally located genes associated with antibiotic resistance: an A deletion in the *mtrR* promoter as well as a missense mutation at codon 45 (G45D) in *mtrR*, two missense mutation in *porB* resulting in PorB amino acid changes (G120K) and 121 (A121D), a missense mutation in *ponA* at codon 421 (L421P), missense mutations as *gyrA* codons 91 (S91F) and 95 (D95A), a missense mutation in *parC* (S87R), and A2059G in 23S rRNA.

The identical sequence types between pre- and post-SPC treatment samples obtained from the male suggested a probable SPC treatment failure. Further, because the partner samples were identical using these tests, it is likely that a transmission of this resistant strain occurred between the couple. To determine the genetic basis for SPC resistance in the isolates we sequenced the 16S *rRNA* and *rpsE* genes as previously described [8]. All three isolates contained a wild-type 16S *rRNA* gene, but had a deletion of three nucleotides (TTA) in the *rpsE* gene at position 80–82 compared with that of *N. gonorrhoeae* reference strain FA1090 (GenBank accession no. AE004969.1). Three nucleotide deletion in *rpsE* resulted in a deletion of amino acid 27 (V) and a K28E amino acid alteration in the ribosomal protein S5. A nucleotide blast search found the *rpsE* genes of all three isolates were identical to a high-level SPC-resistant strain previously reported by Unemo (GenBank accession no. KC311362.1) [8].

SPC has been introduced and proved to be effective for the treatment of urogenital and anorectal gonorrhoea for nearly 50 years. Although the discontinuation of SPC manufacturing has restricted its use to a few countries including China, it is still an alternative antibiotic for patients with a cephalosporin or PEN allergy. WHO also recommended SPC as single therapy based on the local antimicrobial susceptibility surveillance data [9]. In China, SPC is readily accessible and recommended by national treatment guideline and widely used for gonococcal infections in China. In a nationwide survey of antibiotic use, 7.9% of 2121 physicians use SPC as the first option for gonorrhoea treatment [3]. The PK/PD data of SPC shows that the antimicrobial effect of SPC is concentration-dependent and *C*_max_ can reach 100 mg/L 1 h after 2 g dose intramuscular injection. When *C*_max_/MIC is between 8 and 10 folds, the SPC treatment is considered to be effective. As the MIC of the resistant strains are as high as 2048 mg/L, higher doses of SPC within a safe level or CRO should be considered as an appropriate treatment to effectively control spreading of the SPC-resistant clone in China and beyond. Although SPC resistance may emerge quite quickly when it is routinely used for gonorrhoea treatment, the emergence and transmission of a high-level SPC-resistant clone, which seemed to have occurred with the male and female pair studied herein, were sporadic worldwide [8,10] and has not heretofore been reported in China since 2012. Notably, the isolates reported here were only sensitive to CRO and expressed resistance to multiple antibiotics including AZM. Although uncommon, the dual SPC and AZM resistance expressed by the strains described in this report coupled with their possible spread might compromise the dual therapy strategy recommended by WHO in cases where AZM and CRO fail to cure the infection [9]. Accordingly, a strengthened and continuous national surveillance in China is necessary to detect the spread of SPC-resistant isolates of *N. gonorrhoeae*.

## Ethics

The project has been approved by the Medical Ethics Committee at the Institute of Dermatology, the Chinese Academy of Medical Sciences & Peking Union Medical College, and the National Center for Sexually Transmitted Disease Control (NCSTD) at Nanjing (approval number 2014-LS-026).
